# The Influence of COVID‐19 on the Composition and Drug Resistance of Pleural Effusion in Shandong Province

**DOI:** 10.1002/mbo3.70285

**Published:** 2026-04-12

**Authors:** Wenwen Yu, Shuqing Ma, Fen Xu, Xiangmin Han, Ming Li, Yuanqi Zhu, Sufei Pan, Sujun Hou, Chunqing Ma, Fawen Deng, Shifu Wang, the SPARSS net Working Group, Ningning Ge, Fen Su, Renzhe Li, Fengyan Pei, Chunhua Han, Chengjie Guo, Mingju Hao, Bin Ji, Yan Sun, Xiulei Xue, Mingyan Sun, Yilei Li, Hongyun Cao, Hong He, Fang Wang, Zheng Zhou, Xiaoling Zan, Weiping Zhou, Xuejiao Leng, Chunzhong Dong, Xu Zheng, Shiying Sun, Wei Li, Yongjuan Ma, Jin Li, Xiao‐ying Li, Hongyan Bi, Jinjing Tian, Zhiyong Zhang, Ruifang Yang, Yulong Dai, Xuerong Sun, He Yi, Bo Yu, Fengzhi Bian, Qin Yi, Haiying Chen, Qingling Liu, Guixia Li, Jinfang Guo, Hongning Zhang, Huili Hou, Tao Chen, Zhiwei Liu, Ping Wu, Jiajia Feng, Li‐e Zhang, Junjie Dai, Jie Chen

**Affiliations:** ^1^ Department of Clinical Microbiology Jinan Children's Hospital Jinan China; ^2^ Department of Clinical Microbiology Children's Hospital Affiliated to Shandong University Jinan China; ^3^ Clinical Laboratory Center, Weihai Municipal Hospital, Cheeloo College of Medicine Shandong University Weihai China; ^4^ Department of Clinical Laboratory Zhangqiu District Traditional Chinese Medicine Hospital of Jinan City Jinan China; ^5^ Department of Clinical Laboratory JiNan ZhangQiu district maternal and child health hospital Jinan China; ^6^ Department of Clinical Laboratory The Affiliated Hospital of Qingdao University Qingdao China; ^7^ Department of Clinical Laboratory Shengli Oilfield Central Hospital Dongying China; ^8^ Department of Clinical Laboratory Rizhao Municipal Hospital of Traditional Chinese Medicine Rizhao China; ^9^ Department of Clinical Laboratory People's Hospital of Jinan Zhangqiu district Jinan China; ^10^ Department of Hospital Infection Control Dong ‘e County People's Hospital, Dong ‘e Liaocheng China; ^11^ Affiliated Hospital of Jining Medical University Jining Shandong China; ^12^ Weifang People's Hospital Weifang Shandong China; ^13^ Jining NO.1 People's Hospital Jining Shandong China; ^14^ Jinan Central Hospital affiliated to Shandong University Jinan Shandong China; ^15^ The Affiliated Hospital of Qingdao University Qingdao Shandong China; ^16^ Zibo Central Hospital Zibo Shandong China; ^17^ Shandong Qianfoshan Hospital Jinab Shandong China; ^18^ Binzhou medical university hospital Yantai Shandong China; ^19^ Zaozhuang Municipal hospital Zaozhuang Shandong China; ^20^ Liaocheng People's Hospital Liaocheng Shandong China; ^21^ The Affiliated Hospital of Shandong Second Medical University Jinan Shandong China; ^22^ People's Hospital of Rizhao Rizhao Shandong China; ^23^ Zibo Linzi district People's Hospital Zibo Shandong China; ^24^ Central People's Hospital of Tengzhou Zaozhuang Shandong China; ^25^ Shandong Public Health Clinical Center Jinan Shandong China; ^26^ Affiliated Hospital of Shandong University of Traditional Chinese Medicine Jinan Shandong China; ^27^ Women and Children's Health Care Hospital of Lingyi Linyi Shandong China; ^28^ Weihai Central Hospital Weihai Shandong China; ^29^ Binzhou People's Hospital Binzhou Shandong China; ^30^ Qingdao Municipal Hospital Qingdao Shandong China; ^31^ Qilu Hospital of Shandong University (Qingdao) Qingdao Shandong China; ^32^ Dongying People's Hospital Dongying Shandong China; ^33^ Lanling People's Hospital Linyi Shandong China; ^34^ The Second Affiliated Hospital of Shandong First Medical University Jinan Shandong China; ^35^ Weifang Traditional Chinese Hospital Weifang Shandong China; ^36^ Heze Municipal Hospital Heze Shandong China; ^37^ The Second People's Hospital of Liaocheng Liaocheng Shandong China; ^38^ Qilu Hospital of Shandong University Dezhou Hospital Jinan Shandong China; ^39^ Yantai Affiliated Hospital of Binzhou Medical University Shandong China; ^40^ Qingdao Women and Children's Hospital Qingadao Shandong China; ^41^ Caoxian People's Hospital Caoxian Shandong China; ^42^ Yantaishan Hospital Yantai Shandong China; ^43^ The Fourth People's Hospital of Jinan Jinan Shandong China; ^44^ Zouping People's Hospital Binzhou Shandong China; ^45^ Maternal Child Health Care of Zaozhuang Zaozhuang Shandong China; ^46^ Zibo Maternal and Child Health Hospital Zibo Shandong China; ^47^ Tancheng People's Hospital Linyi Shandong China; ^48^ Binzhou second People's Hospital Binzhou Shandong China; ^49^ Zhaoyuan People's Hospital Zhaoyuan Shandong China; ^50^ Liaocheng Dongchangfu district maternal and child health hospital Liaocheng Shandong China; ^51^ Jinan Maternal and Child Health Hospital Jinan Shandong China; ^52^ Taian Maternity and Child Care Hospital Taian Shandong China; ^53^ Changqing District People's Hospital of Jinan City Jinan Shandong China; ^54^ Weifang Maternal and Child Health Hospital Weifang Shandong China; ^55^ Ningjin People's Hospital Dezhou Shandong China; ^56^ Women & Children's Health Care Hospital of Huantai Zibo Shandong China; ^57^ Shandong Nanshan Hospital Jinan Shandong China

**Keywords:** coagulase‐negative staphylococci, contamination, COVID‐19, drug resistance rate, pleural effusion

## Abstract

To analyze the composition and drug resistance changes of pleural effusion in Shandong region from 2017 to 2024, and to provide a basis for clinical empirical treatment and future public health strategies. Uese the WHONET5.6 software to analyze the data reported by the SPARSS network. A total of 6336 pathogens was isolated, 3876 were Gram‐positive bacteria and 2211 were Gram‐negative bacteria. The top five pathogens are *Staphylococcus aureus*, *Staphylococcus epidermidis*, *Klebsiella pneumoniae*, *Streptococcus constellatus*, and *Escherichia coli*. The main pathogens in male patients are *S. constellatus*, *S. epidermidis*, and *K. pneumoniae*, while in female patients, they are mainly *S. aureus*, *S. epidermidis*, and *E. coli*. The detection rate of *S. constellatus* rose from 4.3% to 8.1% after the COVID‐19 pandemic (*p* < 0.0001); *K. pneumoniae* rose from 6.9% to 8.9%(*p* = 0.0264), and its resistance rate to meropenem increased from 2.1% to 18.3% (*p* = 0.0063). The detection rate of methicillin‐resistant *Staphylococcus aureus* has remained largely unaffected by the COVID‐19. The resistance rate of *Candida albicans* to fluconazole is as high as 12.5%. The pathogen spectrum of pleural effusion in Shandong Province is mainly Gram‐positive bacteria. Coagulase‐negative staphylococci were among the most common isolates, yet their role as true pathogens versus culture contaminants remains to be clarified in clinical practice. Moreover, after the epidemic, the carbapenem resistance of *K. pneumoniae* has significantly increased, and the problem of fungal drug resistance also needs to be paid attention to continuous monitoring to guide the rational use of drugs in clinical practice.

## Graphical abstract

Our results show that the COVID‐19 pandemic has a significant impact on the detection of pathogens and drug resistance in pleural effusion in Shandong Province.

## 1 Introduction

Pleural effusion (PE), defined as the pathological accumulation of fluid in the pleural cavity, is a common complication of respiratory diseases and carries considerable clinical significance (Jany and Welte 2019). It is estimated that more than 1.5 million patients are affected annually in the United States alone (Tian et al. 2021). The etiology of pleural effusion is diverse. Notably, when parapneumonic effusions become infected, they may progress to empyema, a condition associated with significantly elevated mortality (Merchant and Liu 2024; Chan et al. 2023). Epidemiological studies indicate that a consistent upward trend in the incidence of pleural infections. During the second decade of the 21st century, the reported incidence in North America, Western Europe, and East Asia rose to 6.7–9.9 cases per 100,000 population, nearly doubling compared to the previous decade (Xu et al. 2022). This trend underscores pleural infection as a growing clinical concern.

It is noteworthy that the global pandemic of Coronavirus Disease 2019 (COVID‐19) has further exacerbated this challenge. COVID‐19 not only imposes a significant public health burden, but severe cases can lead to lung tissue damage, immune dysfunction, and the need for invasive ventilation, which may increase the risk of secondary bacterial pneumonia and pleural infections in affected patients (Zhang et al. 2020; Wiersinga et al. 2020). In response to potential concurrent or secondary bacterial infections in COVID‐19 patients, broad‐spectrum antibiotics have been widely used empirically in clinical practice. While this approach helps control infection, it has also contributed to the global issue of inappropriate antimicrobial use and exerted strong selective pressure on pathogenic bacteria (Langford et al. 2023; Tang et al. 2023).

Currently, there is a paucity of systematic assessments regarding the dynamic changes in the microbial composition of pleural effusion pathogens and their antimicrobial resistance during the COVID‐19 pandemic. Our study aims to elucidate the shifts in pathogen composition and the evolving trends of antibiotic resistance across three distinct periods: pre‐pandemic (2018–2019), during the pandemic (2020–2022), and post‐pandemic (2023–2024). Utilizing retrospective analysis of pathogen detection data from pleural effusion samples collected in Shandong Province between 2017 and 2024, we aspire to provide scientific evidence that can inform the optimization of clinical pathways for pleural infections in the post‐pandemic era, enhance the precision management of antibiotics, develop effective resistance prevention strategies, and guide the formulation of public health policies.

## 2 Materials and Methods

### 2.1 Collection of Strain Sources

The study included 59 participating medical institutions, consisting of 52 tertiary hospitals and 7 secondary hospitals, collectively forming the Shandong Province Pediatric Antimicrobial Resistance Surveillance System (SPARSS) net from January 1, 2017 to December 31, 2024, over an 8‐year period, and repeated isolated strains from the same patient were excluded.

All pleural effusion specimens were collected following standard sterile procedures to minimize skin and environmental contamination. Only samples meeting clinical and laboratory criteria for true infection (e.g., consistent with empyema, positive direct microscopy, clinical response to targeted therapy) were prioritized for analysis, although complete exclusion of contaminating coagulase‐negative staphylococci (CoNS) cannot be guaranteed in a retrospective surveillance study.

### 2.2 Methods

#### 2.2.1 Bacterial Identification

Strain identification was carried out using matrix‐assisted laser desorption/ionization time‐of‐flight mass spectrometry, API systems, and fully automatic microbial identification systems, and so on.

#### 2.2.2 Drug Sensitivity Test

Drug sensitivity tests are conducted using E–test, MIC method, disk diffusion method, and so on. Drug sensitivity tests were conducted with VITEK 2‐Compact, WALKWAY‐96 and other compatible drug sensitivity cards, and so on.

#### 2.2.3 Judgment Criteria

Refer to The Clinical and Laboratory Standards Institute (CLSI) M100‐ED35 (CLSI 2025), M27M44S‐Ed3 CLSI 2022a and the European Committee on Antimicrobial Susceptibility Testing M57S‐Ed4 CLSI 2022b is used to determine the drug susceptibility results.

#### 2.2.4 Definitions Before, During, and After the COVID‐19 Pandemic

Before the COVID‐19 pandemic, 2017–2019; During the COVID‐19 pandemic, 2020‐2022; After the COVID‐19 pandemic, 2023‐2024.

#### 2.2.5 Age Groups

Children < 18 years old, adults > = 18 years old.

#### 2.2.6 Data Analysis

Data analysis was conducted using WHONET5.6 software and GraphPad Prism 8.0 software. The *χ²* test was used to compare the drug resistance rate and species composition of bacteria. A *p*‐value < 0.05 was considered statistically significant.

## 3 Results

### 3.1 Overall Pathogen Distribution

Between 2017 and 2024, member institutions of the SPARSS network isolated a total of 6336 pathogens from pleural effusion samples. Among these isolates, Gram‐positive bacteria constituted 61.2% (3876/6336), Gram‐negative bacteria accounted for 34.9% (2211/6336), fungi represented 3.2% (201/6336), and anaerobes comprised 0.6% (40/6336). Pathogens derived from children patients accounted for 3.8% (238/6336) of the total, predominantly consisting of Gram‐positive bacteria (80.7%, 192/238), followed by Gram‐negative bacteria (16.4%, 39/238), fungi (2.5%, 6/238), and anaerobes (0.4%, 1/238). In contrast, pathogens from adult patients comprised 96.2% (6098/6336), with Gram‐positive bacteria constituting 60.4% (3684/6098), Gram‐negative bacteria at 35.6% (2172/6098), fungi at 3.2% (195/6098), and anaerobes at 0.6% (39/6098). Among the 201 fungal isolates identified, the five most prevalent species were *Candida albicans* (39.3%, 79/201), *Candida tropicalis* (16.4%, 33/201), *Candida glabrata* (13.4%, 27/201), *Candida parapsilosis* (12.4%, 25/201), and *Aspergillus fumigatus* (4.0%, 8/201).

**Figure 1:**
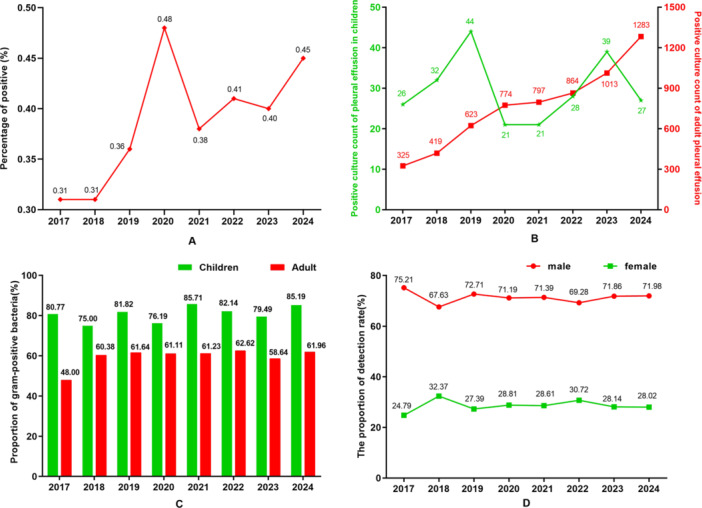
Distribution of pathogens detected in pleural effusion from 2017 to 2024. (A) The proportion of pathogens detected in pleural effusion among all isolated strains. (B) The number of strains isolated from pleural effusion in adults and children. (C) The proportion of Gram‐positive bacteria in pleural effusion of adults and children. (D) The proportion of male and female isolates pathogens in pleural effusion.

The proportion of pleural effusion pathogens among the total pathogen isolates reported by the SPARSS network remained stable at 0.31% to 0.36% prior to the COVID‐19 pandemic. Following the onset of the pandemic, this proportion increased, peaking at 0.48% in 2020, before stabilizing around 0.4% thereafter (see Figure 1A). Since 2017, the annual number of pathogen isolations from adults has shown a steady increase, with respective strains of 325, 419, 623, 774, 797, 864, 1,013, and 1,283; in contrast, the annual number of isolates from children has exhibited relatively minor fluctuations, reporting 26, 32, 44, 21, 21, 28, 39, and 27 isolates (See Figure 1B). Notably, the proportion of Gram‐positive bacteria among pediatric pleural effusion pathogens ranged from 75.0% to 85.71%, significantly higher than that observed in the adult group, which ranged from 48.0% to 62.62% (See Figure 1C). Furthermore, male patients consistently dominated the detection of pathogens in pleural effusions, with proportions stabilizing between 67.63% and 75.21% (see Figure 1D).

From 2017 to 2024, the top five detected pathogens in pleural effusion specimens from the SPARSS network were *Staphylococcus aureus* (8.3%, 523/6336), *Staphylococcus epidermidis* (7.9%, 503/6336), *Klebsiella pneumoniae* (7.4%, 469/6336), *Streptococcus constellatus* (7.3%, 463/6336), and *Escherichia coli* (7.0%, 443/6336). Compared to pre‐pandemic levels, there was a significant decrease in the detection rates of *S. aureus* and *Enterobacter cloacae* following the pandemic, with statistically significant differences observed (*p* < 0.05). In contrast, the detection rates of *K. pneumoniae*, *Pseudomonas aeruginosa*, and *Streptococcus intermedia* showed marked increases, also with statistically significant differences (*p* < 0.05). It is notable that the detection rate of *S. constellatus* rose from 4.3% before the COVID‐19 pandemic to 8.1% after the pandemic (*p* < 0.0001) (see Table 1).

**Table 1:** The changes of the top 15 detected pathogens in pleural effusion before, during, and after the COVID‐19 pandemic.

Pathogens	2017–2024	B	D	A	*p* value		
	(*N* = 6336)	(*N* = 1469)	(*N* = 2505)	(*N* = 2362)	B vs D	D vs A	B vs A
*S. aureus*	523 (8.3)	145 (9.9)	189 (7.5)	189 (8.0)	0.0107*	0.5518	0.0462*
*S. epidermidis*	503 (7.9)	107 (7.3)	204 (8.1)	192 (8.1)	0.33	0.9847	0.3432
*K. pneumoniae*	469 (7.4)	101 (6.9)	158 (6.3)	210 (8.9)	0.4837	0.0007*	0.0264*
*S. constellatus*	463 (7.3)	63 (4.3)	209 (8.3)	191 (8.1)	< 0.0001*	0.7443	< 0.0001*
*E. coli*	443 (7.0)	118 (8.0)	174 (6.9)	151 (6.4)	0.2051	0.4397	0.0534
*E. faecium*	320 (5.1)	67 (4.6)	124 (5.0)	129 (5.5)	0.5798	0.4219	0.2187
*S. hominis*	308 (4.9)	62 (4.2)	127 (5.1)	119 (5.0)	0.2246	0.9597	0.2462
*P. aeruginosa*	305 (4.8)	61 (4.2)	107 (4.3)	137 (5.8)	0.8572	0.0146*	0.0251*
*A. baumannii*	218 (3.4)	67 (4.6)	69 (2.8)	82 (3.5)	0.0025*	0.1493	0.09
*S. haemolyticus*	199 (3.1)	55 (3.7)	81 (3.2)	63 (2.7)	0.3928	0.2439	0.0607
*S. anginosus*	190 (3.0)	50 (3.4)	77 (3.1)	63 (2.7)	0.5683	0.3963	0.1902
*E. faecalis*	146 (2.3)	37 (2.5)	60 (2.4)	49 (2.1)	0.8076	0.4498	0.3668
*E. cloacae*	145 (2.3)	46 (3.1)	53 (2.1)	46 (1.9)	0.0474*	0.6777	0.02*
*S. intermedius*	145 (2.3)	17 (1.2)	56 (2.2)	72 (3.0)	0.0145*	0.0766	0.0002*
*S. mitis*	134 (2.1)	36 (2.5)	48 (1.9)	50 (2.1)	0.2582	0.6184	0.4977

*Note:* B: Before the COVID‐19 pandemic, 2017–2019. D: During the COVID‐19 pandemic, 2020–2022. A: After the outbreak of COVID‐19, 2023–2024.

* Indicate < 0.05

### 3.2 The Comparison of the Top Five Pathogenic Bacteria Isolated From Different Populations

**Figure 2:**
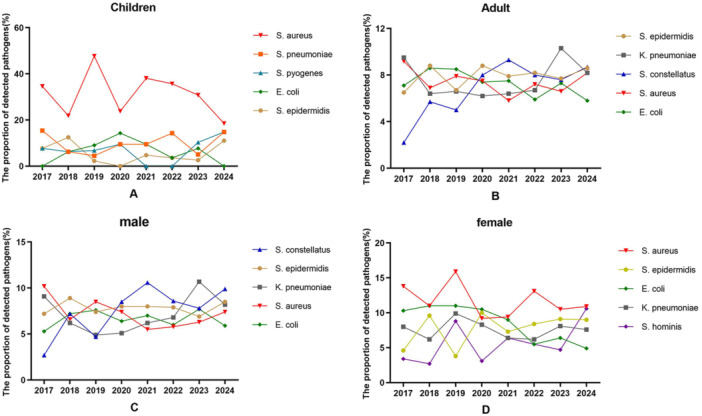
The changes of the top five pathogenic bacteria isolated from pleural effusion among different populations from 2017 to 2024. (A) Changes in the proportions of the top five pathogens detected in children's pleural effusion in different years. (B) Changes in the proportions of the top five pathogens detected in adult's pleural effusion in different years. (C) Changes in the proportions of the top five pathogens detected in pleural effusion of male patients in different years. (D) Changes in the proportions of the top five pathogens detected in pleural effusion of female patients in different years.

The top five pathogens isolated from the pediatric pleural effusions from 2017 to 2024 were as follows: *S. aureus* (32.4%, 77/238), *Streptococcus pneumoniae* (9.2%, 22/238), *Streptococcus pyogenes* (7.1%, 17/238), *E. coli* (6.3%, 15/238), and *S. epidermidis* (5.5%, 13/238). (See Figure 2 A) In adults, the leading pathogens identified in pleural effusions were *S. epidermidis* (8.0%, 490/6098), *K. pneumoniae* (7.6%, 465/6098), *S. constellatus* (7.5%, 455/6098), *S. aureus* (7.3%, 446/6098), and *E. coli* (7.0%, 428/6098). Notably, the proportion of *S. constellatus* increased significantly from 2.2% in 2017% to 8.7% in 2024 (*χ²* = 15.10, *p* < 0.0001) (see Figure 2B). Regarding gender distribution, a total of 4521 pathogen strains were isolated from male patients. The primary pathogens included *S. constellatus* (8.1%, 367/4521), *S. epidermidis* (7.9%, 355/4521), *K. pneumoniae* (7.3%, 332/4521), *S. aureus* (7.0%, 316/4521), and *E. coli* (6.7%, 301/4521) (See Figure 2C). In female patients, a total of 1,815 pathogen strains were isolated, with the top five pathogens being *S. aureus* (11.4%, 207/1815), *S. epidermidis* (8.2%, 148/1815), *E. coli* (7.8%, 142/1815), *K. pneumoniae* (7.5%, 137/1815), and *Staphylococcus hominis* (6.2%, 113/1815). (See Figure 2D) There are significant differences in the pathogen spectrum composition of *S. constellatus* between male and female patients.

### 3.3 Changes in the Drug Resistance Rate of the Mainly Pathogen Detected in Pleural Effusion

#### 3.3.1 Changes in Drug Resistance Rates of *S. aureus* Before, During, and After the Epidemic

From 2017 to 2024, *S. aureus* isolated from pleural effusion specimens by member institutions of the SPARSS network exhibited a high rate of resistance to penicillin, reaching 92.7%. The resistance rates to erythromycin and clindamycin were 67.7% and 48.8%, respectively; no strains resistant to vancomycin or linezolid were detected. Compared to pre‐pandemic levels, there was a significant reduction in the resistance rates of *S. aureus* to gentamicin and tetracycline following the pandemic, decreasing from 17.3% to 7.0% and from 22.2% to 8.8%, respectively (*p* < 0.05). Among all *S. aureus* strains sourced from pleural effusions, methicillin‐resistant *S. aureus* (MRSA) accounted for 30.0%. There were no significant fluctuations in the MRSA proportion across the pre‐pandemic, pandemic, and post‐pandemic periods, with respective proportions of 29.5%, 29.9%, and 30.5% observed (See Table 2).

**Table 2:** Changes in the drug resistance rate of *Staphylococcus aureus* isolated from pleural effusion before, during and after the COVID‐19 pandemic.

Antibiotic	2017–2024		B		D		A		*p* value		
	N	R%	N	R%	N	R%	N	R%	B vs D	D vs A	B vs A
Linezolid	515	0	139	0	187	0	189	0	—	—	—
Vancomycin	513	0	141	0	187	0	185	0	—	—	—
Rifampicin	513	1.8	139	1.4	187	2.1	187	1.6	0.6418	0.7028	0.9043
Gentamicin	511	9.6	139	17.3	185	6.5	187	7	0.0022*	0.8577	0.0037*
Moxifloxacin	477	10.9	126	14.3	177	9.6	174	9.8	0.2089	0.9582	0.2292
Tetracycline	417	14.4	126	22.2	143	13.3	148	8.8	0.0541	0.2196	0.0019*
Ciprofloxacin	432	14.6	127	18.1	155	12.9	150	13.3	0.2262	0.9114	0.274
Levofloxacin	489	15.1	132	16.7	177	14.7	180	14.4	0.635	0.9477	0.5909
Cotrimoxazole	507	20.9	136	17.6	184	23.9	187	20.3	0.1756	0.4044	0.5469
Oxacillin	513	30	139	29.5	187	29.9	187	30.5	0.9299	0.9103	0.8479
Clindamycin	506	48.8	139	56.1	180	42.8	187	49.2	0.0181*	0.2174	0.2163
Erythromycin	511	67.7	139	77	184	59.2	188	69.1	0.0008*	0.0462*	0.1171
Penicillin G	510	92.7	138	94.2	185	90.3	187	94.1	0.1987	0.1664	0.9742

*Note:* B: Before the COVID‐19 pandemic, 2017‐2019. D: During the COVID‐19 pandemic, 2020‐2022. A: After the outbreak of COVID‐19, 2023‐2024.

* Indicate < 0.05.

#### 3.3.2 Changes in Drug Resistance Rates of Cons (Mainly S. epidermidis) Before, During, and After the COVID‐19 Pandemic

*S. epidermidis* exhibited high resistance rates to penicillin G, erythromycin, and levofloxacin, recorded at 93.1%, 76.7%, and 61.4%, respectively. In contrast, the resistance rates to vancomycin and linezolid were notably low, at 0.0% and 0.4%, respectively. Following the pandemic, there was a significant increase of 6.3% in the resistance rate of *S. epidermidis* to penicillin G compared to during the pandemic (*p* < 0.05). (See Table 3).

**Table 3:** Changes in the drug resistance rate of *Staphylococcus epidermidis* isolated from pleural effusion before, during, and after the COVID‐19 pandemic.

Antibiotic	2017–2024		B		D		A		*p‐*value		
	N	R%	N	R%	N	R%	N	R%	B vs D	D vs A	B vs A
Vancomycin	490	0	104	0	198	0	188	0	—	—	—
Linezolid	490	0.4	103	0	198	0.5	189	0.5	0.47	0.9737	0.4596
Rifampicin	485	7.6	94	7.4	201	9.5	190	5.8	0.5712	0.1738	0.5896
Gentamicin	488	13.3	104	13.5	195	12.8	189	13.8	0.8754	0.787	0.9439
Tetracycline	419	22	86	29.1	174	21.8	159	18.2	0.2005	0.4131	0.0509
Moxifloxacin	479	26.3	102	25.5	192	26	185	27	0.9181	0.8285	0.7776
Clindamycin	488	45.9	104	47.1	194	42.3	190	48.9	0.4216	0.1889	0.7638
Ciprofloxacin	433	57.5	100	56	172	55.2	161	60.9	0.9023	0.2977	0.4368
Cotrimoxazole	493	59	102	64.7	200	60.5	191	54.5	0.4765	0.2264	0.0902
Levofloxacin	484	61.4	103	60.2	196	58.7	185	64.9	0.7993	0.2141	0.4308
Oxacillin	496	75.6	104	76	201	71.6	191	79.6	0.4199	0.0677	0.4711
Erythromycin	490	76.7	104	78.8	195	75.9	191	76.4	0.5644	0.9005	0.6374
Penicillin G	493	93.1	103	93.2	201	90	189	96.3	0.3602	0.0152*	0.2373

*Note:* B: Before the COVID‐19 pandemic, 2017‐2019. D: During the COVID‐19 pandemic, 2020‐2022. A:After the outbreak of COVID‐19, 2023‐2024.

* Indicate < 0.05

#### 3.3.3 Changes in Drug Resistance Rates of *K. Pneumoniae* Before, During, and After the Epidemic

*K. pneumoniae* demonstrated resistance rates of 19.9%, 32.6%, 33.3%, and 22.8% to ceftazidime, ceftriaxone, cefotaxime, and aztreonam, respectively. The resistance rate to cefepime was 18.5%, while the resistance rates to imipenem, meropenem, and ertapenem were 10.2%, 7.6%, and 8.6%, respectively. Compared to pre‐pandemic levels, there was a significant increase in the resistance rates to amoxicillin‐clavulanate, cefepime, imipenem, meropenem and ertapenem following the pandemic (*p* < 0.05), especially, the resistance rate to meropenem increased from 2.1% to 18.3% (*p* = 0.0063) (see Table 4).

**Table 4:** Changes in the drug resistance rate of *K. pneumoniae* isolated from pleural effusion before, during, and after the COVID‐19 pandemic.

Antibiotic	2017–2024		B		D		A		*p* value		
	N	R%	N	R%	N	R%	N	R%	B vs D	D vs. A	B vs. A
Tigecycline	157	3.8	18	0	55	3.6	84	4.8	0.412	0.7495	0.3449
Meropenem	210	7.6	48	2.1	72	5.6	93	18.3	0.3511	0.015*	0.0063*
Ertapenem	315	8.6	68	1.5	106	5.7	141	14.2	0.17	0.0307*	0.0042*
amikacin	403	8.7	82	6.1	143	5.6	178	12.4	0.8763	0.0385*	0.124
Imipenem	403	10.2	82	4.9	141	6.4	180	15.6	0.6437	0.0106*	0.0144*
Piperacillin/tazobactam	400	13.2	83	9.6	143	9.1	174	18.4	0.8913	0.0182*	0.0703
Tobramycin	325	15.4	67	17.9	110	10.9	148	17.6	0.187	0.1356	0.9513
Cefoperazone/Sulbactam	268	15.7	60	10	78	14.1	130	19.2	0.4673	0.3439	0.1095
Cefepime	411	18.5	82	12.2	144	16.7	185	22.7	0.3659	0.175	0.0455*
Ceftazidime	376	19.9	69	17.4	123	14.6	184	24.5	0.6137	0.0368*	0.2309
Gentamicin	356	20.8	80	25	119	16.8	157	21.7	0.1573	0.3145	0.5617
Amoxicillin/Clavulanic acid	184	21.2	22	4.5	72	22.2	90	24.4	0.0594	0.7401	0.0383*
Aztreonam	372	22.8	80	18.8	124	20.2	168	26.8	0.8042	0.19	0.1672
ESBL	279	24	66	21.2	88	25	125	24.8	0.5826	0.9735	0.5785
Cotrimoxazole	408	27.9	82	28	140	29.3	186	26.9	0.8443	0.632	0.8432
Levofloxacin	379	29.6	82	24.4	126	27	171	33.9	0.6767	0.2015	0.1245
Ceftriaxone	359	32.6	73	32.9	125	28.8	161	35.4	0.547	0.237	0.7066
Cefotaxime	171	33.3	27	40.7	56	33.9	88	30.7	0.545	0.6837	0.331
Cefuroxime	225	35.6	52	34.6	76	38.2	97	34	0.683	0.5733	0.9419
Ampicillin/sulbactam	323	35.9	68	30.9	98	30.6	157	41.4	0.9704	0.083	0.1359
Ciprofloxacin	339	39.2	80	37.5	110	37.3	149	41.6	0.9745	0.4807	0.5452
Cefazolin	193	53.8	41	56.1	71	43.7	81	61.8	0.2045	0.0259*	0.549

*Note:* B: Before the COVID‐19 pandemic, 2017‐2019. D: During the COVID‐19 pandemic, 2020‐2022. A: After the outbreak of COVID‐19, 2023‐2024.

* Indicate < 0.05.

#### 3.3.4 Changes in the Resistance Rate of *S. Constellation* Before, During and After the Epidemic

S. constellation has a relatively high resistance rate to erythromycin and clindamycin, which are 80.1% and 85.3% respectively. Its resistance rate to penicillin is relatively low, at 0.5%. Its resistance rates to vancomycin and linezolid are 0.0% and 0.3% respectively. After the epidemic, compared with during the epidemic, the resistance rate of *S. constellation* to levofloxacin has decreased significantly. However, its resistance rate to clindamycin increased significantly (*p* < 0.05) (see Table 5).

**Table 5:** Changes in the drug resistance rate of *Streptococcus constellation* isolated from pleural effusion before, during and after the COVID‐19 pandemic.

Antibiotic	2017–2024		B		D		A		*p*‐value		
	N	R%	N	R%	N	R%	N	R%	B vs D	D vs. A	B vs. A
Vancomycin	344	0	39	0	156	0	149	0	—	—	—
Linezolid	296	0.3	38	0	136	0.7	122	0	0.596	0.3426	—
Penicillin G	367	0.5	39	0	168	0	160	1.2	—	0.1461	0.4828
Chloramphenicol	185	2.2	20	5	85	2.4	80	1.2	0.5226	0.5962	0.284
Levofloxacin	322	2.8	37	0	147	5.4	138	0.7	0.1468	0.0228*	0.6036
Cefotaxime	186	7	22	0	85	5.9	81	7.4	0.244	0.693	0.1884
Erythromycin	346	80.1	36	72.2	159	78	151	84.1	0.4585	0.1702	0.0967
Clindamycin	348	85.3	35	85.7	165	81.2	148	89.9	0.5289	0.0309*	0.4791

*Note:* B: Before the COVID‐19 pandemic, 2017‐2019. D: During the COVID‐19 pandemic, 2020‐2022. A:After the outbreak of COVID‐19, 2023‐2024.

* Indicate < 0.05

### 3.4 Sensitivity Analysis of Major Fungi in Pleural Effusion to Common Antifungal Drugs

The resistance rate of *Candida albicans* to fluconazole in pleural effusion was 12.5%, while the resistance rates of *Candida tropicalis* and *Candida glabrata* were relatively high, at 25.0% and 20.0% respectively. Meanwhile, according to the epidemiological critical value, in the drug sensitivity results of *C. tropicalis* to itraconazole, the proportion of non‐wild types was relatively high, reaching 25.9%. The resistance rate of *Candida parapsilosis* to fluconazole is relatively low, accounting for only 5.9% (see Table 6).

**Table 6:** Sensitivity analysis of major fungi in pleural effusion to common antifungal drugs.

Fungi	Amphotericin B			Fluconazole			Voriconazole			Itraconazole		
	N	S/WT(%)	R/NWT(%)	N	S/WT(%)	R/NWT(%)	N	S/WT(%)	R/NWT(%)	N	S/WT(%)	R/NWT(%)
*C. albicans*	63	100%*	0.0%*	64	84.4%	12.5%	65	83.0%	10.8%	—	—	—
*C. tropical*	26	100.0%*	0.0%*	28	75.0%	25.0%	30	43.3%	26.7%	27	74.1%*	25.9%*
*C. glabrata*	20	100.0%*	0.0%*	20	—	20.0%	22	68.2%*	31.8%*	20	95.0%*	5.0%*
*C. parapsilosis*	17	100.0%*	0.0%*	17	94.1%	5.9%	18	88.8%	5.6%	16	93.8%*	6.2%*

*Note:* WT: Wild type. NWT: Non‐Wild type.

* Represents wild type and non‐wild type as determined by ECV. ‐ indicates no data due to the absence of sensitive breakpoints

## 4 Discussion

Pleural infections represent a serious complication of respiratory diseases, with persistently high rates of morbidity and mortality observed over time (Abdulelah and Abu Hishmeh 2024; Corcoran et al. 2020; Markatis et al. 2022; Brims et al. 2019). The onset of the COVID‐19 pandemic has further exacerbated the challenges associated with the clinical management of pleural infections. Studies have shown that patients with accompanying pleural effusion often present with more severe clinical symptoms and elevated inflammatory markers, such as C‐reactive protein (CRP) and procalcitonin (PCT). Increases in both CRP and PCT are typically indicative of poor prognosis and are associated with greater disease severity and an increased risk of mortality (Rathore et al. 2022). Additionally, pleural infection can lead to localized structural changes that affect the penetration of antimicrobial agents, making it difficult for certain drugs (e.g., aminoglycosides) to achieve effective therapeutic concentrations at the site of infection, thereby increasing the risk of treatment failure (Lau et al. 2022). Simultaneously, the continual rise in the detection rates of Gram‐negative bacteria, particularly the widespread emergence of resistant strains, has further complicated treatment efforts and heightened patient mortality risks (Assefa 2022; Brink 2019). Against this backdrop, early and accurate pathogen identification, along with the rational selection of empirical antibiotic therapy, has become crucial for improving patient outcomes.

To systematically evaluate the impact of the COVID‐19 pandemic on the distribution of pathogens and resistance characteristics in pleural effusion, this study analyzed data from 6,336 pathogen reports submitted by 59 member institutions within the Shandong Province SPARSS surveillance network from 2017 to 2024. The results indicate that adults comprise the primary demographic for pathogen detection in pleural effusions (96.2%), which aligns with epidemiological data showing a high prevalence of pleural effusion among adults in China (Tian et al. 2021). In terms of etiological distribution, parapneumonic effusion, empyema, malignancy, and tuberculosis are identified as the main causes of pleural effusions in adults. Notably, parapneumonic effusion and empyema are particularly prevalent in the age groups of 40‐59 years and those aged 80 years and older (Tian et al. 2021). Further analysis reveals that adverse lifestyle habits (such as smoking and alcohol consumption) and underlying health conditions (including chronic obstructive pulmonary disease and diabetes) are significant risk factors associated with the occurrence and poor prognosis of empyema. This underscores the importance of enhancing comprehensive health interventions and managing underlying diseases in clinical practice to reduce the incidence risk in affected populations (Merchant and Liu 2024).

In terms of pathogen composition, Gram‐positive bacteria remain dominant in this study, accounting for 61.2%, consistent with previous research (Chan et al. 2023; Foley and Parrish 2023). Coagulase‐negative staphylococci (CoNS), especially S. epidermidis and S. hominis, were among the most frequently isolated organisms. In surveillance studies without real‐time clinical adjudication, it is challenging to fully distinguish pathogenic isolates from contaminants, which may lead to overestimation of the etiological role of CoNS in pleural infections. However, a significant change in the microbial structure has occurred following the COVID‐19 pandemic. Specifically, the detection rate of *S. aureus* decreased from 9.9% pre‐pandemic to 8.0% (*χ²* = 3.975, *p* < 0.05). In contrast, the prevalence of methicillin‐resistant *S. aureus* (MRSA) remained stable throughout the study period, ranging between 29.5% and 30.5%. This finding is in agreement with the systematic review by Bradley J. Langford et al (Langford et al. 2023), suggesting that there is no direct correlation between the COVID‐19 pandemic and changes in MRSA case proportions. Nonetheless, MRSA, as a pathogen widely resistant to β‐lactam antibiotics, continues to be one of the leading drug‐resistant organisms associated with high mortality rates globally. As of 2024, it remains classified as a high‐priority pathogen for intervention by the World Health Organization (Yan et al. 2025). Its robust biofilm‐forming ability facilitates colonization and persistent infection within the pleural cavity, often leading to treatment difficulties, increased mortality rates, and prolonged hospital stays (Cheung et al. 2021; Kaushik et al. 2024). Therefore, the ongoing reinforcement of hospital infection control measures and resistance surveillance is crucial for curbing MRSA transmission and improving patient outcomes.

In this study, *S. epidermidis* ranked as the second most prevalent pathogen (7.9%) among all isolated organisms from pleural effusion specimens. As the most common species among CoNS, *S. epidermidis* is typically a commensal bacterium colonizing the skin, nasal cavity, and mucosal surfaces (Burke et al. 2024). However, in recent years, it has gradually evolved into an important opportunistic pathogen associated with healthcare‐associated infections (Heilmann et al. 2019). Its pathogenicity primarily relies on its capacity for biofilm formation and the host immune status, rendering it one of the key pathogens in infections related to implantable medical devices (Burke et al. 2024; Michalik et al. 2020). Antimicrobial susceptibility analysis revealed that *S. epidermidis* exhibited high resistance rates to penicillin G (93.1%), erythromycin (76.7%), and oxacillin (75.6%). Notably, the resistance rate to penicillin G in the post‐pandemic period increased by 6.3% compared to the intra‐pandemic period (*p* < 0.05), indicating persistent antimicrobial selective pressure. In contrast, the bacterium remained highly susceptible to vancomycin and linezolid, suggesting that glycopeptides and oxazolidinones continue to be effective options for treating confirmed CoNS infections (Ye et al. 2022). It is noteworthy that linezolid‐resistant CoNS strains have been reported in recent years (Gostev et al. 2021), warranting clinical vigilance regarding the further evolution of resistance. When CoNS are cultured from clinical specimens, a comprehensive assessment integrating the patient's underlying diseases, history of medical exposure, and clinical presentation is essential. This approach is crucial both to avoid misinterpreting contamination as infection, which could lead to unnecessary antibiotic use, and to prevent treatment delays caused by overlooking CoNS as a genuine pathogen.

A noteworthy finding in this study is the significant increase in the detection rate of *S. constellatus* after the pandemic, rising from 4.3% pre‐pandemic to 8.1% (*χ²* = 21.10, *p* < 0.0001). This change may be attributed to the widespread use of immunosuppressive medications, such as dexamethasone, Janus kinase inhibitors, and inflammatory cytokine blockers, in critically ill COVID‐19 patients. These treatments can impair host immune function, thereby increasing the risk of opportunistic infections (Abdoli et al. 2022). Additionally, the application of new technologies, including metagenomic next‐generation sequencing, has enhanced the sensitivity of clinical detection for *S. constellatus* (Zhu et al. 2022), leading to a gradual increase in reported cases of related empyema (Xia et al. 2021; Zhu et al. 2024). Research indicates that empyema caused by *S. constellatus* is particularly common among elderly male patients with diabetes, oral infections, or a history of oral surgery. Management often requires a comprehensive approach that includes pleural drainage, nutritional support, intravenous antibiotics, and, in some cases, surgical intervention (Lin et al. 2022).

Regarding Gram‐negative bacteria, *K. pneumoniae* has emerged as the predominant pathogen post‐pandemic, with its detection rate increasing from 6.9% pre‐pandemic to 8.9% (*χ²* = 4.932, *p* = 0.0264). Of particular concern is the significant rise in the proportion of carbapenem‐resistant *K. pneumoniae* (*CRKP*), with resistance rates to imipenem and meropenem escalating from 4.9% to 15.6% (*χ² *= 5.990, *p* = 0.0144) and from 2.1% to 18.3% (*χ²* = 7.458, *p* = 0.0063), respectively. As a class of extensively drug‐resistant “superbugs,” *CRKP* has been classified as an urgent public health threat by the Centers for Disease Control and Prevention (Yang et al. 2022). The rising trend in resistance rates aligns with multiple studies conducted both domestically and internationally (Du et al. 2025; Echegorry et al. 2024), likely linked to the overuse of broad‐spectrum antibiotics during the COVID‐19 pandemic (Allel et al. 2023) and the horizontal transmission of resistance genes. Data from a hospital in Chile indicated that the intensity of carbapenem use surged from 50.9 to 110.1 during the peak of the pandemic, while the infection rate of *CRKP* carrying the *bla*NDM gene rose from 40% to 73.6% (Allel et al. 2023). In China, approximately 60% of *CRKP* isolates carry the *bla*KPC gene, with the ST11 clone predominating, often resulting in nosocomial transmission and outbreaks (Ge et al. 2024).

The overall detection rate of fungi in this study was relatively low (3.2%), mainly *Candida albicans*. Fungal empyema is relatively rare in clinical practice and often occurs secondary to chest surgery, trauma, abdominal cavity infection or esophageal perforation, etc (Senger et al. 2021). Although the detection rate is not high, its mortality rate is often higher than that of bacterial empyema (Cheng et al. 2023). Therefore, early identification and standardized antifungal treatment are particularly important. echinocandin drugs (such as micafungin and caspofungin) are usually used as first‐line options, while azole drugs (such as fluconazole and voriconazole) can be used for combination or monotherapy. Amphotericin B is often used as an alternative for severe infections, and close monitoring of renal function and electrolyte levels is required during use (Lui et al. 2021). This study shows that the resistance rate of *C. albicans* to fluconazole is as high as 12.5%. In previous studies, the resistance rate of *C. albicans* to fluconazole has generally remained at a low level (Bilal et al. 2022; Xiao et al. 2020; Wang et al. 2024). This study observed an increase in the resistance rate of *C. albicans* to fluconazole, which requires high vigilance in clinical practice. However, due to the limited number of *C. albicans* detected in this study, the sample size still needs to be further expanded to clarify its drug resistance characteristics and epidemic trend.

This study also has several limitations. Firstly, it relied on traditional bacterial culture and antimicrobial susceptibility testing without incorporating molecular diagnostic or serological methods. This may have resulted in under‐detection of fastidious microorganisms or difficult‐to‐culture pathogens. Secondly, as a retrospective surveillance study, we could not systematically adjudicate whether CoNS isolates represented true infection or sample contamination; this may have led to overestimation of their clinical significance in the pathogen spectrum. Thirdly, the monitoring period following the pandemic (2023–2024) is relatively short; thus, the observed changes in the pathogen profile and antimicrobial resistance may be difficult to categorize as short‐term fluctuations or long‐term trends. Ongoing surveillance will be necessary to clarify these observations further.

In conclusion, this study systematically analyzed the impact of the COVID‐19 pandemic on the composition of pleural infection pathogens and the trend of drug resistance through long‐term and large‐sample regional surveillance of pleural effusion etiology and drug resistance. CoNS were frequently isolated but require careful clinical interpretation to avoid overinterpretation of contaminants. In the post‐pandemic era, clinical practice should pay attention to the characteristics of pathogen spectrum changes, dynamically optimize empirical anti‐infection plans based on epidemiological data, continuously strengthen etiological and drug resistance monitoring, strictly implement infection control measures, comprehensively enhance the comprehensive diagnosis and treatment capabilities of thoracic infections, effectively delay the spread of drug‐resistant bacteria, and provide support for building a more resilient medical and health system.

## Author Contributions

**Wenwen Yu:** conceptualization, investigation, methodology, data curation, software, formal analysis, validation, writing – original draft, visualization. **Shuqing Ma:** data curation. **Fen Xu:** data curation. **Xiangmin Han:** data curation. **Ming Li:** data curation. **Yuanqi Zhu:** data curation. **Sufei Pan:** data curation. **Sujun Hou:** data curation. **Chunqing Ma:** data curation. **Fawen Deng:** data curation. **Shifu Wang:** data curation, methodology, conceptualization, funding acquisition, supervision, writing – review and editing, visualization, project administration, validation, resources.

## Ethics Statement

All experiments involving human participants were performed according to the guidelines and regulations of the Declaration of Helsinki (2013 version). The study guarantees that the identities of the participants and other related data have been kept anonymous and confidential. The requirement for informed consent was waived because of the retrospective nature of the study.

## Conflicts of Interest

The authors declare no conflcits of interest.

## Data Availability

The data that support the findings of this study are available from the corresponding author upon reasonable request.
